# Low-Field NMR for Carbon-Modified Cements: Dispersion and Hydration Studies

**DOI:** 10.3390/ma19030528

**Published:** 2026-01-29

**Authors:** Mihai M. Rusu, Karoly Mostis, Codrut Costinas, Ioan Ardelean

**Affiliations:** 1 Department of Physics and Chemistry, Technical University of Cluj-Napoca, 400114 Cluj-Napoca, Romania; mihai.rusu@phys.utcluj.ro (M.M.R.);; 2 EUT+ Institute of Nanomaterials & Nanotechnologies EUTINN, European University of Technology, European Union, 64295 Darmstadt, Germany; 3 Faculty of Physics, Babeș-Bolyai University, M. Kogălniceanu 1, 400084 Cluj-Napoca, Romania; 4 Laboratory for Advanced Materials and Applied Technologies, Institute for Research, Development and Innovation in Applied Natural Sciences, Babeș-Bolyai University, Fântânele 30, 400294 Cluj-Napoca, Romania

**Keywords:** carbon black, cement, hydration, multifunctional concrete, NMR relaxometry

## Abstract

**Highlights:**

**What are the main findings?**

- Improved colloidal stability of carbon black into water is revealed in the presence of an acrylic-based superplasticizer at SP/CB weight ratios between 0.1 and 2.
- During the in situ NMR mixing of carbon ink with cement, the peak ascribed to the carbon ink decreases with water content and paste mixing time.
- In fresh cement pastes, an increase in superplasticizer dosage induced smaller initial transverse relaxation times and slower evolutions in the relaxation rate, indicating improved dispersion of cement particles and slower structural build-up.
- In hardened cements, the increase in superplasticizer dosage induced more narrow relaxation time distributions and shorter relaxation times, indicators of a more refined pore network.

**What are the implications of the main findings?**

- They open new approaches in the characterization of carbon-containing cementitious composites that are non-contact and compatible with hydrated and hardened paste samples and allow a lower consumption of carbon materials during research stages.
- They further clarify the impact of the carbon black-dispersant role over cement hydration and microstructure.
- They contribute to the developing research interface between fundamental studies on cement, multifunctional carbon-integrated composites, and fields including smart buildings and additive manufacturing.

**Abstract:**

This study investigates the interface between cement hydration, low-field NMR relaxometry, and the incorporation of carbon-based fillers into cementitious materials. The objective is to provide NMR-based insights into how carbon black (CB) and an acrylic superplasticizer (SP) influence cement hydration and the resulting microstructural evolution. CB was integrated into white Portland cement (WPC) using both wet and dry mixing approaches, with water content and SP dosage varied independently. First, water-based “inks” containing different SP/CB weight ratios were prepared and evaluated through dynamic light scattering (DLS) and ζ-potential measurements to assess colloidal stability and dispersibility. For the wet-mixing route, an in situ NMR experiment was performed to monitor the progressive incorporation of carbon ink into cement pastes while increasing the water content. The ability to distinguish ink-related signals from those originating from the cement paste represents a promising step toward non-destructive assessments of carbon dispersion in fresh pastes. Separately, ex situ NMR measurements were performed on samples extracted from dry-mixed pastes with various SP dosages. These experiments mark the SP-induced delay in hydration and the refinement of the pore network that is also associated with improved particle dispersion. Complementary optical microscopy (OM) and ultrasonic pulse velocity (UPV) measurements on hardened samples corroborate the NMR findings.

## 1. Introduction

Cement is the world’s most widely used man-made construction material, serving as the fundamental component of modern infrastructure. In mortar and concrete, mixtures based on water and cement function as binders, obtained via a complex hydration process [1]. Upon curing, these composites achieve their characteristic high mechanical strength and durability. Historically, supplementary additives have been incorporated to enhance performance characteristics such as workability, setting time, and final strength.

An emerging and significant paradigm in material science involves embedding conductive fillers into the cement matrix, a development that moves beyond traditional structural goals [2]. Since bulk concrete is naturally an electrical insulator, the introduction of such materials can transform the composite into a smart, multifunctional material. Owing to its improved electrical and thermal transport behaviors, including pronounced piezoresistive and thermoelectric responses, carbon-modified cement can be engineered to serve multifunctional roles that extend well beyond traditional structural performance [3]. The resulting applications might include structural health monitoring (self-sensing materials able to detect strain, stress, and damage) [4], advanced capabilities for detecting and autonomously healing micro-cracks [5], self-heating systems [6], or 3D printing [7], as well as energy harvesting [8] and energy storage in construction elements [9].

Among the various conductive fillers explored, carbon-based additives have earned substantial attention due to their exceptional electrical conductivity and chemical stability within the alkaline cement environment. This broad class of fillers encompasses materials ranging from a macro to a nano scale and from amorphous to crystalline, mentioning here only the widely studied forms such as carbon black (CB), carbon fibers, graphite flakes, carbon nanotubes, and graphene [10]. While some carbon allotropes (e.g., carbon nanotubes) may offer superior electrical and mechanical behavior, CB is employed in more accessible formulations of cement composites with potential applicability in self-sensing [5], self-heating [11], and energy storage [9]. It is produced by partial pyrolysis of organic compounds and is particularly attractive due to its relatively high conductivity, reduced health hazards, low cost, and wide availability [3]. However, issues regarding workability and dispersibility, lower interfacial bonding ability, and a compromise between mechanical and electrical performance are highlighted across the literature [12]. In this context, dispersants and superplasticizers (SPs) play a critical role in achieving a uniform distribution and maximizing performance [11,12,13]. The recent literature has laid a foundation for understanding carbon-modified cementitious systems, carbon dispersion levels, and the effective tools to quantify them. For instance, Zhang et al. systematically explored the influence of various dispersants on the performance of fresh carbon black-filled cement, focusing primarily on its macroscopic rheology and hardened properties [13]. D’Alessandro et al. utilized empirical coefficients to quantify carbon nanotube dispersion in the presence of different surfactants, relying on ink stability tests and SEM micrographs to corroborate percolation and strain-sensing measurements [14]. Chanut et al. used correlative Raman/EDS imaging for tracking the carbon network formed within the cement matrix and electrochemical measurements for addressing the energy storage and the scalability of carbon–cement-based capacitors [9]. However, composite optimizations in terms of structure, morphology, filler concentration, and level of dispersion still represent a significant step and remain a time-consuming challenge [13,15]. Non-destructive and time-efficient tools able to track the life cycle of carbon additives from a pre-mixed “ink” into a solid cement structure remain limited. Thus, it is highly relevant to identify alternative techniques able to accelerate research in similar fields.

Aiming at the intersection between NMR, carbon, and cement research, we have noticed a gap in the experimental works that we propose to address in the present study. Low-field NMR relaxometry is a robust, non-contact, non-destructive technique frequently used in the analysis of a diverse palette of ^1^H rich systems. One key advantage is the ability to probe liquids found in bulk states or confined in various environments such as pore networks, capsules, and loose pastes. In cement studies with low-field NMR techniques, a wide spectrum of information can be gathered regarding the chemical and physical processes taking place during the entire hydration process. Different parameters such as the hydration degree [16,17,18], fractal dimensions [19], and mass transport properties for different confined molecules, as well as their porosity and pore size distribution [20,21,22], were determined for different cement systems. Similarly, NMR techniques are used in the study of carbon materials as well. Investigations were reported on various microporous and mesoporous carbon materials [23], carbon aerogels and xerogels [24,25,26,27], nano-CB, graphene, and nanotubes [28,29]. The studies relied on relaxometry or diffusion techniques able to probe the hydrophilic or hydrophobic nature and the fractal size and tortuosity of the pore networks found in monolithic samples. One other approach is to investigate the dispersions of functionalized carbon nanotubes and their aggregation tendency in water and anionic polyelectrolytes, as shown by Porelli et al. [30]. Huang et al. [28] successfully applied NMR to characterize carbon nanotube (CNT) solutions in their fluid state, prior to cement embedding. Still, according to our knowledge, the transfer of these carbon-based fillers into the cement matrix remains under-explored.

Our study addresses this gap by introducing a novel “process-monitoring” paradigm that treats the additive (a mixture of water, CB, and SP) as a discrete, complex fluid “ink”. By first isolating and evaluating the transverse relaxation response of these dense inks, the methodology provides a quantitative benchmark for CB dispersion quality prior to its introduction into the cementitious environment. Furthermore, rather than confining the analysis to a single water-to-cement ratio (w/c), this research monitors the incorporation of the ink across a progressively higher w/c gradient.

Based on previous work on printable mortars and on porous carbon materials, we anchor the current research using three focal points: (1) evaluating the NMR response of dense “inks” based on water, CB, and SP; (2) monitoring the incorporation of the ink into cement at progressively higher w/c values; and (3) monitoring the effect of SP dosage on the cement hydration, pore network development, and CB dispersion using the CPMG sequence to determine transverse relaxation time distributions. Stability and dispersion tests based on dynamic light scattering (DLS) and ζ-potential measurements, plus optic microscopy (OM) investigations and ultrasonic pulse velocity (UPV) tests on hardened samples complement the present NMR results. One should note that in the multifunctional CB-modified cements and mortars used, i.e., for self-heating, sensing, or energy storage, promising results were obtained for a CB content ranging from 5 to 10% by cement mass, while the SP dosages and w/c ratios were selected to be between 1 and 4% and 0.4 and 0.8, respectively [3,9,31]. Based on this, we focused on paste formulations that remain feasible in terms of mixing, casting, and extruding, narrowing the scope to cement pastes containing 5–10% CB (by cement weight) and 0–2% SP.

## 2. Materials and Methods

### 2.1. Materials

White Portland cement (WPC) CEM I 52.5 R (Holcim, Bucharest, Romania), fulfilling the European Standard EN197-1 [32], carbon black, acetylene powder (ThermoFisher Scientific, Waltham, MA, USA), and an acrylic-based superplasticizer (Dynamon, Mapei, Milan, Italy) were used for sample preparation. The bulk density and specific surface area of CB are reported by the manufacturer as 170–230 g/L and 75 m^2^/g, respectively. Cyclohexane (Merck, Darmstadt, Germany) was used as a probing molecule during desorption experiments.

The oxide composition (in wt%) of WPC was determined as 70.7 (CaO), 16.3 (SiO_2_), and 4.7 (Al_2_O_3_), with gypsum-derived sulfate (5.8%) and 0.9 (MgO), 0.7 (Na_2_O), 0.6 (K_2_O), and 0.3 (Fe_2_O_3_) as impurities [33]. The low iron oxide content reduces internal gradient effects on the transverse relaxation measurements and thus provides a more reliable determination of the relaxation time distributions, while the white appearance of the cement improves the optical contrast between the matrix and the electron-conductive filler. No metal impurities (apart from Al originating from the substrate) were observed in the elemental composition of CB. One should note that in the present context, the cement chemistry abbreviations are used, where C = CaO, S = SiO_2_, A = Al_2_O_3_, F = Fe_2_O_3_, and H = H_2_O, so that, i.e., CSH refers to the calcium silicate hydrate phase [34].

### 2.2. Sample Preparation

The in situ experiment, in which the water content is progressively increased, is designed to optimize paste consistency while minimizing sample volume. The tested scenario corresponds to the production of a cement paste with a high CB content (10% by weight of cement) and an average SP dose (2% by weight of cement). For the in situ NMR experiment, a small quantity of carbon “ink” was prepared inside an NMR tube. The carbon ink composition was selected so that SP/CB = 0.2 and W/CB = 3(without including the water fraction from the commercial SP). An ultrasonic bath treatment was applied for 15 min in an ice–water mixture and left to stabilize at 22 °C before the first CPMG measurements. The next step consisted of mixing the ink with the entire mass of the anhydrous cement powder for 1 min and measuring the NMR response. A water-to-cement ratio of 0.3 was selected as the starting point for the CB–cement mix. This was followed by 10 steps consisting of a water increase, a 1 min mix, and NMR measurement. Water content was adjusted so that the w/c of the paste increased by an increment of 0.1 before each measurement. Mixing was performed on a vibrating table and the experiment was performed at 22 °C.

A second experiment was designed with the purpose of investigating the NMR response of CB, containing fresh pastes prepared in large volumes. The starting context is based on tuning paste consistency (and CB dispersion) by adjusting the SP dose. It should be noted that to enhance paste mixability both in the presence and absence of the superplasticizer a relatively high water-to-cement ratio (w/c = 0.8) was adopted, while the carbon black content was reduced to 5 wt% of cement, compared to 10 wt% used in the in situ measurements. A dry mixing approach was chosen for the preparation of the fresh cement pastes: WPC and CB dry powders were homogenized for 10 min. After this, the water–superplasticizer solution was added. The SP content varied between 0 and 1 wt% (by cement mass). The resulting pastes were mixed for 5 min using an electric mixer.

For the NMR and microscopy investigations, the cementitious pastes were cast into NMR tubes (diameter 8.5 mm, height 20–30 mm) and vibrated for 15 s on a vibrating table. The tubes were sealed for a period of 28 days. Afterwards, the samples were removed from the tubes, cut, and polished to obtain cylindrical samples with an 8 mm diameter and a 15 mm height suitable for NMR studies on cyclohexane-saturated samples. The resulting compositions are later denoted as Dp, where “p” represents the SP content (wt% by cement mass).

### 2.3. Characterization Techniques

#### 2.3.1. Dispersion, Stability, and DLS ζ-Potential Tests

The dispersibility and stability of the carbon black–water mixture was initially evaluated by visual inspection after ultrasonic treatment in the water–ice mixture for different periods between 5 and 90 min. The water-to-carbon black weight ratio was set to 500.

Diluted CB–SP aqueous mixtures were freshly prepared to determine hydrodynamic particle size distribution and colloidal stability. A Malvern Nano ZS90 Zetasizer particle analyzer (Malvern Panalytical, Worcestershire, UK) equipped with a He–Ne laser (633 nm, 5 mW) was used to examine the particle size distributions and the ζ-potential of the aqueous colloidal solutions of each sample, which were subjected to an ultrasonic bath to ensure deagglomeration. Each investigation consisted of 5 sets of 12 measurements, all taken at a scattering angle of 90° and at a temperature of 25 °C. The laser attenuation level for each measurement was chosen automatically by the software. The water-to-carbon black weight ratio was adjusted to 2500.

#### 2.3.2. NMR Relaxometry Experiments

Transverse relaxation time (T_2_) distributions were obtained using the Carr–Purcell–Meiboom–Gill (CPMG) pulse sequence [35]. Briefly, a train of π RF pulses follow an initial π/2 pulse, generating a series of echoes recorded at times t = 2nτ (n = 1, 2, 3, …), where 2τ represents the echo time. The echo amplitudes undergo exponential attenuation governed by a transverse relaxation processes. The relaxation time distribution can be further extracted by applying inverse Laplace transforms [36]. If diffusion effects on echo train attenuation can be neglected and pores are fully saturated by the probing liquid [33], the transverse relaxation time of the molecules confined in each pore type can be further linked with their effective surface-to-volume ratio (*S/V*) using Equation (1):


(1) $$\frac{1}{T_{2}}=\frac{1}{T_{2b}}+\rho \frac{S}{V}$$

Here, T_2b_ is the transverse relaxation time for bulk water and ρ is the surface relaxivity parameter characteristic of the effective liquid–pore wall interface. Usually, in cement materials, one assumes T_2b_ >> T_2_. Under certain conditions, during dormancy or hardening stages, relaxivity can also be considered a composition-specific constant parameter [37] so that any variations in T_2_ can be explained in terms of capillary pore morphology.

The NMR measurements were performed with a Bruker Minispec MQ20 spectrometer (Bruker Biospin GmbH, Karlsruhe, Germany) operating at a proton resonance frequency of 20 MHz.

During the in situ investigation of carbon ink incorporation in cement paste, the CPMG trains consisted of 1000 echoes. The scan number was set at 32 for fast measurements. The operating temperature was set at 25 °C. To hinder diffusion effects, one measurement was performed with an echo time of 0.08 ms and a recycle delay of 0.5 s. To detect long relaxation times, another measurement was performed using an echo time of 1.00 ms and a recycle delay of 5 s.

During ex situ hydration studies, the CPMG measurements consisted of sequences of 1000 echoes using echo time intervals of 0.08 ms and a recycle delay of 2 s, the operating temperature being 25 °C. To increase the signal-to-noise ratio, 128 measurements were averaged. Under such conditions, only the signals specific to mobile water were detected (relaxation times as low as ~10^−1^), while diffusion effects on echo train attenuation were minimal.

For sample evaluation under cyclohexane saturation, the cylindric samples obtained after an 8-week hardening period were vacuum dried. Then, saturation was obtained by immersing the samples in cyclohexane-filled vials for 5 days at room temperature. Before CPMG measurements, the samples were removed and roughly tapped with paper to remove only the excess liquid. The samples were inserted and sealed inside NMR tubes to minimize any unwanted solvent evaporation during measurements. The measurements were performed at an operating temperature of 35 °C, using 2000 pulse sequences, a recycle delay of 7 s, and 32 repetitions for averaging. Two echo times of 0.08 ms and 1.00 ms were considered.

#### 2.3.3. Optic Microscopy

To evaluate the effects of the superplasticizer on the microporosity and the dispersion of CB into the cement matrix, longitudinal sections of the composite samples were analyzed. The core of the samples was exposed by polishing. The samples were rinsed with alcohol after each polishing step and left to dry under vacuum conditions.

Investigations were performed using a Leica DM 2500 (Leica Microsystems, Wetzlar, Germany) microscope in dark-field configuration to enhance surface contrast. Images were acquired with 5× and 20× objective lenses. A wide cross-section of the samples was documented by recording a set of 2 × 4 images with the 5× lens across the entire profile of the monoliths.

To obtain an extended depth of field, a series of micrographs at successive z-heights were obtained with a step of 10 μm, when using the 5× lens, and 1 μm in the case of the 20× lens. Each series was combined into a single composite image in FIJI 1.54p software using the extended depth of field plugin [38,39] and analyzed using image segmentation based on computer vision algorithms [40].

#### 2.3.4. Ultrasonic Pulse Velocity (UPV) Tests 

The UPV tests were conducted in accordance with ASTM C597 using a MATEST C372M (Matest S.p.A, Treviolo, Italy) ultrasonic velocity tester. A direct (two-probe) transmission setup was employed, with the transducers having a nominal resonant frequency of 55 kHz. The coupling between the transducers and the specimen surface was ensured using a thin layer of coupling gel. The travel time of the ultrasonic pulse was recorded and the pulse velocity was calculated as the ratio between the specimen length and the measured transit time. All measurements were taken at room temperature and multiple readings were averaged to reduce random errors.

## 3. Results and Discussions

### 3.1. Dispersion and Stability

**Figure 1:**
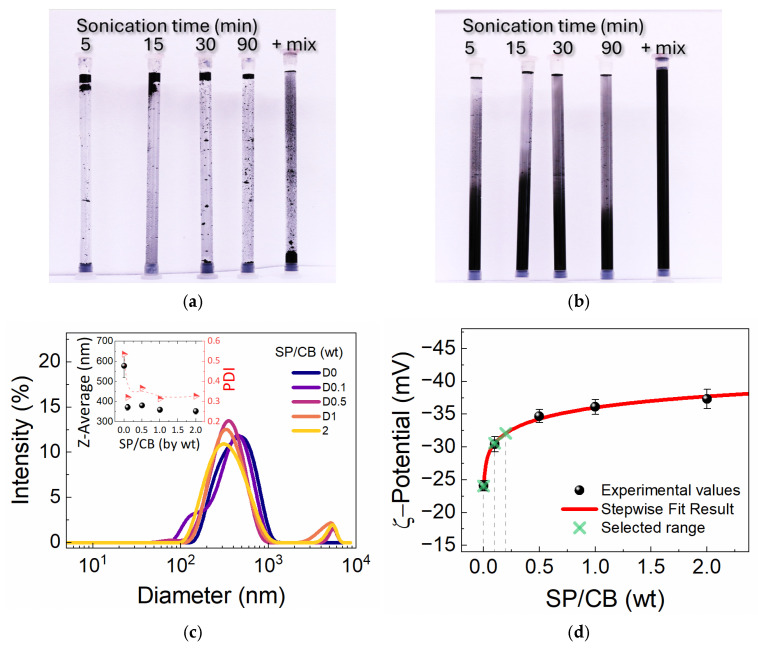
Carbon black (CB) dispersibility and stability in water assessed during visual inspection of sonicated samples of the following: (**a**) plain CB–water mixtures (SP/CB = 0), (**b**) aqueous dispersions at superplasticizer-to-carbon black weight ratios SP/CB = 1, the evolution of (**c**) particle size distribution, (**d**) the ζ-potential at SP/CB ratios from 0 to 2. The inset in (**c**) represents the variation in average size and polydispersity index (PDI) as a function of SP/CB ratio. The green crosses in (**d**) represent the SP/CB ratios selected for hydration studies.

Preliminary dispersion and stability tests were performed to establish the formulation of CB ink that should be further used in the mortar preparation and to evaluate the NMR response characteristics. The results obtained at different SP/CB (weight ratios) are shown in Figure 1. A plain CB–water mix obtained after sonication at different times can be found in Figure 1a. The poor dispersion and mixing can be clearly observed. The result can be further compared to a CB mix with SP/CB = 1 (Figure 1b). The enhanced CB dispersion in the presence of SP was observed for several days, demonstrating a good SP-induced stabilization of CB particles in water.

The particle size distributions were determined at a SP/CB between 0 and 2. The results are summarized in Figure 1c. As the SP/CB increases, one can follow a progressive shift and broadening towards lower dimensions of the primary mode, which is ascribed to CB aggregates. The average aggregate size shown in the inset of Figure 1c exhibits a sharp decrease from 570 ± 60 to 370 ± 15 nm when the SP/CB increases only slightly from 0 to 0.1, followed by a modest decrease to 350 ± 14 nm at SP/CB = 2. The quality of each dispersion was further quantified by the polydispersity index (PDI). The reference sample without the superplasticizer (SP/CB = 0) had a PDI of 0.53 ± 0.06, indicating a broad distribution typical of poorly dispersed, aggregated particles. The introduction of the superplasticizer rapidly improved the suspension’s homogeneity, leading to a PDI reduction across all of the tested ratios (0.1, 0.5, 1, 2), with PDI values around 0.30 ± 0.03 for SP/CB ratios of 1 and 2, demonstrating that the superplasticizer not only reduces the average aggregate size, but also narrows the size distribution, resulting in more uniform and stable suspensions.

Further on, the measured ζ-potentials are presented in Figure 1d. As observed, the magnitudes increase (−36.1 ± 1.2 mV for SP/CB = 1 versus −24.1 ± 0.8 mV for SP/CB = 0) at higher SP dosages, which are above the conventional threshold of |ζ| > 30 mV typically required to establish sufficient electrostatic repulsion for a colloidally stable suspension. The SP polymer chains adsorb onto CB particles until the maximum surface coverage is reached. As suggested by the DLS Zeta measurements, the surface coverage should reach a maximum at SP/CB = 2. The overall results indicate that the superplasticizer effectively balances the attractive forces that drive CB agglomeration.

While electrostatic repulsion is greatly improved with the addition of SP, it is important to mention that the long, adsorbed acrylic polymer chains of the SP most likely induce an additional steric stabilization [41]. The DLS and ζ-potential results clearly demonstrate excellent dispersion under ideal, diluted conditions. However, the dispersions in the high-ionic-strength and crowded environment of the cement paste are significantly more challenging. Therefore, the LF-NMR studies (Section 3.2) further analyze how the effective dispersion of the carbon ink changes during the actual mixing and early hydration of the cement composites.

### 3.2. Low-Field NMR Measurements at Different Hydration Stages

#### 3.2.1. In Situ NMR Tests on the Effect of w/c Ratio

The first empiric framework that can aid in interpreting the NMR findings is provided by qualitative observations of the visual aspect and mixing behavior of the prepared mixtures. Under initial conditions (w/c = 0.3), a poor mixability between cement and carbon ink was observed. Then, both mixability and paste homogeneity improved considerably when the water-to-cement ratio was increased to 0.4. The composition at w/c = 0.5 is reached 25 min after hydration initiation. During mixing, the apparent consistency resembled that of a workable composition for extrusion-based processes. At w/c = 0.8, the paste was still viscous (at 60 min hydration time), while at w/c = 1.0, the mixture was no longer homogeneous, probably due to the occurrence of irreversible hydration reactions.

**Figure 2:**
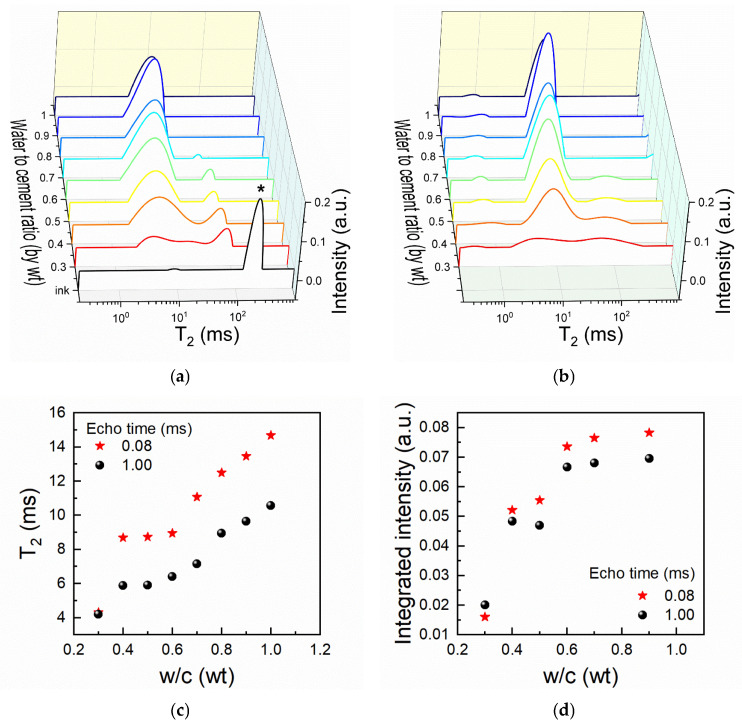
In situ NMR studies on CB “ink” incorporation in fresh cement pastes while mixing and increasing the water to cement ratio: (**a**) Laplace spectra obtained from CPMG measurements with long echo times (1 ms) and (**b**) short echo times (0.08 ms) and the variation of (**c**) T_2_ and (**d**) integrated intensity on capillary water peak. The starred spectrum (*) from (**a**) corresponds to the initial CB “ink” (SP/CB = 0.1) with no cement.

The effect of progressive homogenization and an increase in water content on the transverse relaxation time distribution are presented in Figure 2. Usually, fresh cement paste measurements are performed at short echo times to minimize diffusion effects [42].

However, due to the expected presence of long relaxation times, the current CPMG measurements were performed both at long and short echo times as shown in Figure 2a and Figure 2b, respectively.

Each peak observed in the Laplace spectra can be assigned to water populations confined in different environments. The Laplace spectrum measured for the plain carbon ink is the one marked in Figure 2a. It can be observed that the ink exhibits one characteristic relaxation time at 195 ms and a very small signal at 10 ms. In similar measurements performed on functionalized carbon nanotube suspensions, Porrelli et al. obtained similar T_2_ values [30]. Based on a comparison between water confinement in pore systems, the largest relaxation time was ascribed to the liquid phase where nanotubes were homogeneously distributed, while the smaller T_2_ was ascribed to water confined in highly packed clusters [30]. The first mixture resulting from mixing the cement powder with the carbon ink within the NMR tube is characterized by the red-lined spectra from Figure 2a,b. The mixture corresponds to a paste with w/c = 0.3 and an SP dosage of 2% (wt% by cement mass). The peaks found at relaxation times within 10^0^–10^1^ ms are assigned to water molecules confined in capillary pores of fresh cement pastes [42]. When using short echo times, a second contribution is found within 10^−1^–10^0^ ms, associated with water embedded in cement grain agglomerates and primary hydrates.

Following the transformation of the “CB ink signal” with progressive homogenization and an increase in water content in Figure 2a,b, one observes a clear signal decrease and shift towards lower relaxation times until w/c = 0.8. This can be interpreted based on the empiric observations. During mixing, the carbon ink is sheared and dispersed as progressively smaller cell volumes into the cement paste. As these cells increase their specific surface and interface with the cement paste, faster relaxation mechanisms can be considered to occur due to paramagnetic impurities originating from the cement. The signal disappearance or superposition with the capillary peak at higher w/c ratios is probably due to the fast exchange of water molecules between both the capillary pores and the ink. This effect is enhanced as shearing induces smaller ink volumes and it might be a good indicator that the carbon phase becomes well dispersed into the mass of the cement paste. Even though unconventional, the results obtained recommend the potential use of such NMR measurements for evaluating the degree of mixing between similar fillers and cementitious pastes.

In a previous study, the characteristic parameters of the capillary water peak measured 10 min after hydration initiation were proposed as descriptors able to distinguish between printable and non-printable cement pastes [37]. The initial capillary peak characteristics and paste consistency observed for extrudable mortars based on acrylic superplasticizers and WPC are comparable to those measured for the present CB-modified pastes with w/c ratios of 0.4–0.6. This indicates that printable, carbon-modified compositions may be obtained in this range. Although this lies outside the focus of the present work, the current results allow us to estimate how the initial capillary peak position varies with the water-to-cement ratio and interpret this in terms of effective capillary pore size based on several assumptions. First, the hydration effects are inhibited in presence of the superplasticizer during the early dormancy stage. This is reasonable, considering the relatively high SP dose (2% by weight of cement). The second assumption is that the relaxivity will not be significantly affected during the in situ experiment. The evolution of the relaxation rate with field-cycling-derived pore surface-to-volume ratio was linear during the first ~100 min of the dormancy stage, suggesting that the temperature and surface properties reflected by the relaxivity parameter can be considered to be constants [37]. At present, the extent to which the modification of the capillary pore walls by the superplasticizer-adsorbed carbon black may influence surface relaxivity remains uncertain. Although further investigation is required to address this issue, the present analysis is conducted under the simplifying assumption that such effects are modest. This assumption is supported by the absence of significant paramagnetic impurities (e.g., Fe, Mn) and by the fact that the same superplasticizer is adsorbed on both the carbon black and cement phases.

The transformations of the early capillary peak position and integrated area are represented in Figure 2c,d for both short and long echo time measurements. The observed differences between the two measurements can be explained by diffusion in internal gradients [42,43]. Under the framework described in the previous paragraph, by approximating relaxivity as a composition-specific constant in Equation (1), the observed trends are further interpreted in terms of pore size effects using a percolation model. Initially, water populations are transferred from the carbon ink. At low water contents, the wetting of the solid particles is only partial, which is reflected in the Laplace spectra by poorly defined peaks. Due to the continuous creation of water-filled pore sites (viewed as isolated nodes in a network), both T_2_ and the integrated intensities of the capillary peak increase at high rates between 0.3 and 0.4. Under this regime, a first inflection point is observed close to w/c = 0.35. The capillary pore network becomes connected and the mixture gains sufficient fluidity and mixability to qualify as a paste. Between w/c = 0.4 and 0.6, the connectivity of the capillary pore system increases as more nodes are created. Water populations from different sites including carbon ink and cement pores are more efficiently transferred. This further improves the fluidity and mixability of the paste components. As the experiment continues in the range of w/c = 0.6–0.8, since the network is already highly connected, the transverse time (and pore size) steadily increases. A secondary inflection point appears, probably due to the interconnection between a larger macropore subclass. Future investigations are expected to further substantiate this trend.

#### 3.2.2. NMR and the Effects Introduced by SP Dosage During Early Hydration

**Figure 3:**
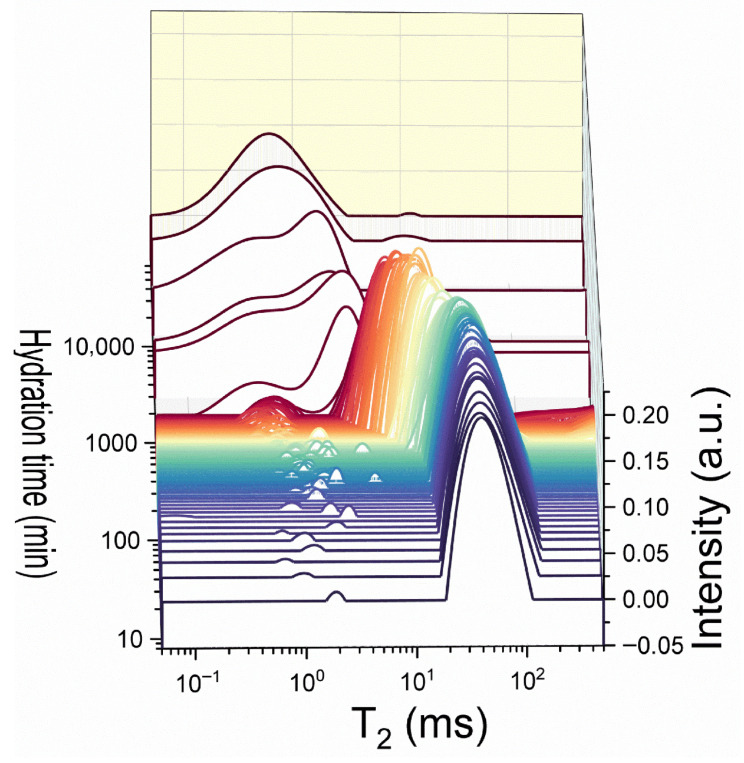
Transverse relaxation time distributions recorded during the full hydration of the D0.5 sample showing the evolution of water in capillary pores, intra-CSH, and inter-hydrate pores.

A representative T_2_ relaxation time distribution obtained for sample D0.5 at different hydration times is shown in Figure 3. As mentioned during the in situ experiment, the two peaks observed during the first hours of hydration are ascribed to the main capillary pores and water embedded within cement grain agglomerates and primary hydrates. At later hydration stages, when capillary water is consumed via hydration reactions, the remanent water is confined in a highly mesoporous network of hydrates. Then, the dominant mode, known as the inter-CSH peak, will be found in the same interval of 10^−1^–10^0^ ms [42]. The smallest relaxation times that are detected around ~10^−1^ ms are usually associated with mobile water diffusing within the lamellar CSH. The distribution peak centered around this value is known as the intra-CSH mode [42]. Others originating from water confined within macropores or from the “carbon ink” could not be detected.

**Figure 4:**
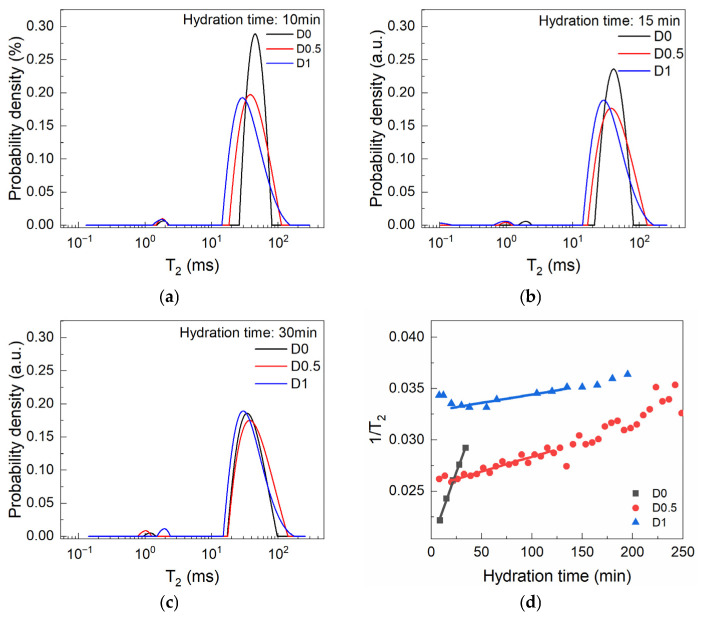
Laplace spectra recorded during the dormancy stage for samples (**a**) D0, (**b**) D0.5, and (**c**) D1 and (**d**) the SP-induced changes over the intervals when the relaxation rates (1/T_2_) show quasi-linear increase.

The Laplace spectra obtained during the first 30 min of hydration for each of the investigated samples are presented in Figure 4. Similarly to the step at w/c = 0.8 of the in situ experiment, the dry-mixed samples do not exhibit a clearly differentiated carbon ink signal. This is attributed mostly to the high w/c ratio and mixing time. However, the capillary peak is centered at larger relaxation times. This is attributed mostly to the lower solid content of the dry-mixed pastes. One may observe that the overall shape of the capillary peak changes in the presence of SP, as broader peaks are observed in the D0.5 and D1 samples. The initial peak position shifts at lower relaxation times, while the integrated intensities increase with SP dosage (wt% by cement mass). The intensity is proportional to the amount of water molecules confined in the entire capillary pore volume analyzed by the detection coils. One should note that the apparent sample volume was fixed for all samples. This may suggest an improved filling of the sampling volume and should be correlated with an increase in paste workability for the SP-containing pastes. As previously discussed, the initial transverse relaxation time associated with the capillary peak is influenced mainly by the pore surface-to-volume ratio as shown in Equation (1). When analyzing Figure 4d, one can observe the linear increase in the relaxation rate. By considering the conditions in which relaxivity is constant, a linear increase in relaxation rate can be interpreted as a linear increase in the surface-to-volume ratio of the capillary pores. While the linearity range can be considered as an NMR equivalent for the open time, the slope of these plots can be correlated with the structural build-up rate of the cement pastes [37]. The increase in SP concentration is shown to generate smaller slopes and thus smaller structuration rates.

The analysis confirms that the chosen superplasticizer plays a dual role in inhibiting cement hydration and improving particle dispersion. These correlate well with the different SP surface coverage of the cement grains combined with an initial Ca^+^ complexation by SP. Indeed, recent studies performed on nano-CB-modified cements and different types and amounts of SP attribute the increased flowability of SP-containing systems with adsorption and steric hindrance effects, probably also coupled with a free water “unwrapping” from CB clusters and ascribe delayed setting to Ca^+^ complexation [13]. Further insights are still necessary to fully understand the complex SP–CB–cement dynamics during the full hydration process.

#### 3.2.3. NMR During the Hardening Stage

**Figure 5:**
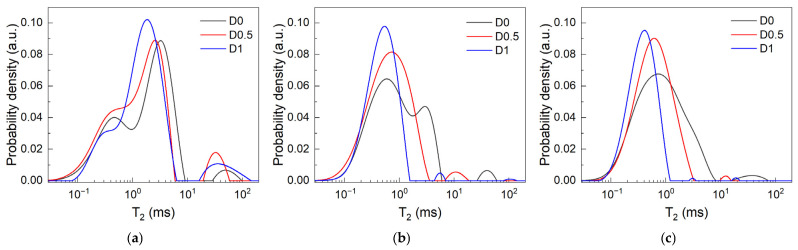
Laplace spectra of the samples containing 0, 0.5, and 1% SP (wt% by cement mass) recorded during the hardening stage at (**a**) 2 days, (**b**) 30 days, and (**c**) 55 days.

Similar measurements are performed during cement hardening to monitor the effects of the superplasticizer on the microstructure. In Figure 5, the Laplace spectra of the three investigated samples are compared at 2, 30, and 55 days after hydration was initiated. In this range, the dominant capillary peak is shifted relative to the position recorded during early stages at relaxation times of 2–5 ms. This is due to the partial filling of the capillary pores and to a pore size reduction given by the growth of the hydrated layer [1]. As the hardening process continues, the capillary mode transits to relaxation times of ~0.5 ms. This value corresponds to the spin relaxation of water molecules confined within the inter-hydrate space, the inter-CSH mode [44].

Following the D0 sample in Figure 5a–c, it can be observed that the peak intensity assigned to partially filled capillary pores drops inversely with the intensity of the inter-hydrate mode, while the peak position is less affected by the hydration process. As an effect, at 55 d, one can observe only a broad and asymmetric T_2_ distribution representing the convolution of the inter- and intra-CSH modes characteristic to cement materials [44]. This feature can indicate the more heterogeneous pore network found in D0.

When analyzing the spectra of the D0.5 and D1 samples from Figure 5a–c, the transformations of the main T_2_ involve lower intensity losses, significant shifts towards lower relaxation times, and narrower distributions as SP content increases. This may be a sign of more refined porous networks.

In the cement hardening stages, if the cement samples are stored in dry conditions, the water from the capillary pores is lost via cement hydration reactions or evaporation. Hence, the previously described NMR analysis lacks any information on the capillary pore system. This limitation can be easily addressed by re-saturating the samples with other solvents including water, ethanol, or cyclohexane [44,45,46]. Due to lower relaxation times, water-saturated samples may generate highly convoluted peaks in their Laplace spectra. Hence, cyclohexane was chosen as a probing molecule as it was previously shown to optimally discriminate the capillary pores from the other contributions due to its non-polar nature and longer relaxation times [44,45,46]. Cyclohexane also exhibits stronger adsorption on carbon surfaces as opposed to water molecules [47].

**Figure 6:**
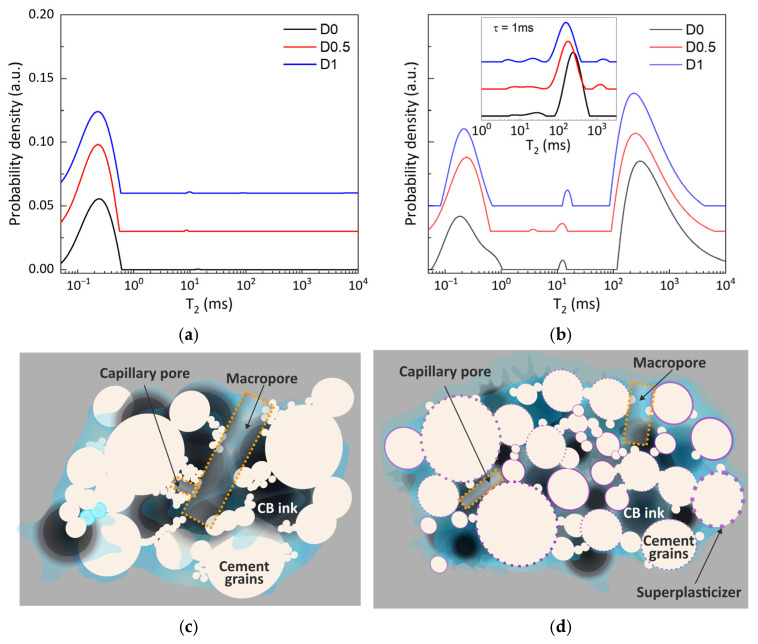
Laplace spectra of (**a**) dried and (**b**) cyclohexane-saturated samples. The inset from (**b**) represents Laplace spectra obtained from CPMG measurements with long echo times (1 ms) and an NMR-based description of pore network development (**c**) in absence and (**d**) presence of SP.

The results of the CPMG measurements performed on the dried and the cyclohexane-saturated samples are shown in Figure 6a,b. Under the present measuring conditions, the intra-CSH peak is observed at 0.2 ms for all samples. Consistently with the previous paragraph, the peak is broader for the D0 sample. The ratio between the intensity of the CSH peaks and the capillary peak (A_CSH_/A_cap_) is 0.34 for D0 and 0.53 and 0.42 for D0.5 and D1, respectively. After saturation with cyclohexane, the capillary peaks are centered at 300 ms for the D0 sample and 250 ms for the SP-containing samples. One can also observe that, at full saturation, all samples exhibit a tail towards longer relaxation times. This feature is enhanced for the D0 sample. When using short echo times of 0.08 ms in the CPMG sequence, the signal decay was measured at 160 ms. Longer relaxation times might not be determined correctly by inverse Laplace transform. Due to this, the interpretation of the observed tail should be performed with caution. To prevent this, supplementary measurements were performed using a longer echo time of 1.00 ms and a longer sequence (2000 ms). These were performed at the expense of introducing diffusion effects. The corresponding Laplace spectra are presented in the inset from Figure 6b. For D0, the main peak is centered at 230 ms and no other significant signals can be observed at larger relaxation times. For samples D0.5 and D1, the main contributions are found at shorter relaxation times of 175 and 160 ms, respectively, but additional peaks can be observed at 1250 and 1400 ms.

The CPMG measurements performed during the early hydration and hardening stages best describe the effects induced by the superplasticizer, as summarized in Figure 6c,d. First, the macropore volume decreases as reflected by the A_CSH_/A_cap_ parameter.

The shorter relaxation times observed for samples D0.5 and D1 correlate with smaller capillary pore size. The tails and the contributions at larger relaxation times may be explained when considering a fast exchange between cyclohexane molecules found in the capillary pores with the ones from larger macropores. Again, these features are more pronounced for the D0 sample and suggest a higher exchange rate between capillary and larger pores (i.e., entrained air bubbles). As illustrated in Figure 6c, this translates to higher contributions of macropores and also higher fractal dimensions and pore network connectivity [19]. An enhanced dispersion of cement and CB particles would reduce macropore size and volume, while larger volumes of CB would fill the capillary pore space. This is characteristic for sample D1 (Figure 6d). Hence, the NMR data obtained from the early stages to a hardened state of CB-integrated cement pastes shows that the presence of superplasticizer refines the entire porous network, mainly due to a more efficient dispersion of WPC and CB particles.

### 3.3. OM and UPV Tests

**Figure 7:**
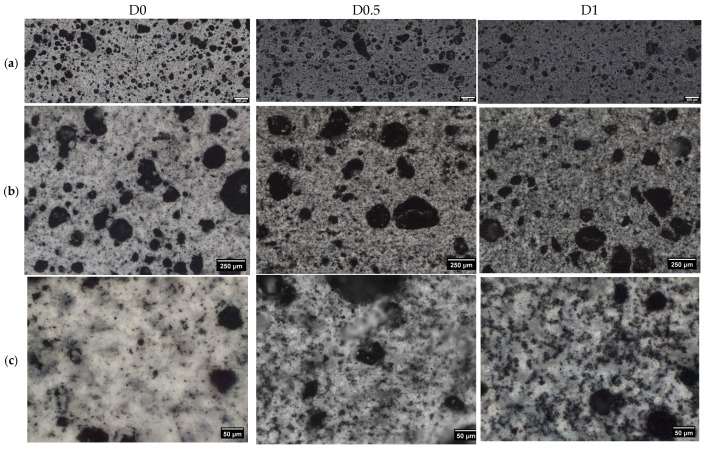
OM micrographs obtained from the cross-section of the samples containing 0, 0.5, and 1% SP (wt% by cement mass) at (**a**) 20×, for CB discrimination; (**b**) 5×, for observations on macropores and phase distributions; (**c**) 5×, 2 × 8 mosaic images sampling the longitudinal cross-section of the samples to evaluate the homogeneity of macropore and color distributions.

The SP effects on the morphology of the composites were further analyzed with optic microscopy (Figure 7). The investigations were performed from lower to higher magnifications, to evaluate the homogeneity and large-scale distribution of each of the composite phases. Three distinct features were observed and automatically discriminated using machine learning algorithms [40]: (1) macropores, (2) carbon aggregates, and (3) features specific to the cement matrix. A 2 × 4 set of low-magnification micrographs was used to scan the cross-section of each sample as represented in Figure 7a. Here, the dark macropores or void structures are well distinguished. The statistical analysis of voids with sizes above 25 μm reveal that the average size decreases from 70 μm for D0 to 67 μm for D1, while the maximum detected size decreases from 465 μm for D0 to 337 μm for D1. The population number of analyzed voids was 700, 520, and 470 for samples D0, D0.5, and D1, respectively. This correlates with the smaller macropore contributions observed for sample D1 in Figure 6b. The image sets also indicate a progressive darkening of the matrix as the superplasticizer dosage increases. This is a direct effect of the improved shearing and redistribution of the carbon ink into smaller CB clusters and probably hyperfine dispersions within the cement matrix. The former features were successfully discriminated during the image segmentation process (Figure 7b,c). Their average size was 3 μm for D0 and D0.5 and 3.5 μm for D1, respectively, which is comparable with typical capillary pore sizes found in cement [48] and the mean chord lengths of CB-forming in cement, as determined by Chanut et al. [9]. The population numbers detected on a surface of 3.24 mm^2^ were 2700, 6300, and 5600 respectively. Future investigations will further expand the multi-scale distribution of CB and its effects on the interaction of CB–cement pore surfaces with various confined liquids.

The UPV values characteristic to the investigated samples were 2400 m/s for D0, 2600 m/s for D0.5, and 2800 m/s for sample D1. The results indicate higher stiffness and mechanical integrity for the D0.5 and D1 samples. As confirmed by the NMR and OM investigations, this can be explained by the SP-induced reduction in macro-porosity and in phase distribution inhomogeneity. One should note that even though the UPV values for normal concrete should surpass the typical threshold of 3500 m/s [49], the presently investigated compositions are not designed for a structural purpose but as a multifunctional composite, with potential in sensing and energy storage applications. In such contexts, the parameters that govern the electric properties (conductivity and capacity) such as the porosity and the carbon distribution play a more important role [9]. Nevertheless, for further optimizations, future studies should also consider the role played by other synthesis parameters such as CB concentration and w/c ratios.

## 4. Conclusions

Carbon inks and composites based on cement, carbon black, and variable concentrations of an acrylic-based superplasticizer were characterized via low-field NMR and complementary techniques.

Promising results were obtained during initial dispersibility and stability tests on aqueous suspensions with superplasticizer-to-carbon black weight ratios between 0.1 and 2. Abrupt shifts in the average hydrodynamic size, PDI, and the ζ-potential of carbon black aggregates were recorded at superplasticizer-to-carbon black weight ratios as low as 0.1. For mixtures with no superplasticizer, an average size of 550 nm and a ζ-potential of −24 mV were recorded, while the lowest, asymptotical limits obtained were 350 nm and −35 mV, respectively, at superplasticizer-to-carbon black weight ratios of 2.

An original in situ NMR experiment was proposed to investigate the progressive dispersion of carbon black in cement pastes with a fixed composition and a progressive increase in mixing and water content. The NMR measurements successfully discriminated water trapped in the “carbon ink” from the water population wetting the cement grains. The experiment emphasizes the decrease in the ink-related signal as the pastes’ mixability and overall homogeneity increases.

The hydration of carbon black-integrated cement pastes was monitored by ex situ NMR studies. The carbon black-to-cement and water-to-cement ratios were fixed at 0.05 and 0.8, respectively, while the superplasticizer dosages tested varied between 0 and 1% (by mass of cement). During early hydration, the increase in superplasticizer dosage shortens the initial values of the main relaxation time attributed to capillary water and its rate of evolution. This is correlated with smaller structuration speed and a more refined pore network. In the hardening stage (2 to 55 days), the NMR signals capture the evolution of intra- and inter-CSH water populations. Measurements on cyclohexane-saturated samples offer more information on capillary pores and macropores, which further supports superplasticizer-induced pore network refinement. The results correlate with the morphological details obtained by optic microscopy and ultrasonic pulse velocity tests.

The present findings highlight the sensitivity of NMR for key aspects concerning multifunctional carbon-modified cement composites. The potential of both in situ and ex situ low-field NMR experiments for characterizing these materials from the first minutes of hydration to hardened states is proven. The approach offers several advantages, including reduced time and material consumption and a non-contact, non-intrusive characterization that does not depend on the sample’s mechanical properties. The interpretations were based on microstructural changes due to shearing and dispersion, under a constant relaxivity approximation. Future work should include more in-depth studies on the effects of CB, superplasticizers, and other admixtures over relaxivity, decoupled from pore size distribution and should further correlate the data with multifunctionality in carbon-modified composites.

## Data Availability

The original contributions presented in the study are included in the article. Further inquiries can be directed to the corresponding author.

## Abbreviations

The following abbreviations are used in this manuscript:

NMR	Nuclear Magnetic Resonance
CB	Carbon Black
SP	Superplasticizer
WPC	White Portland Cement
DLS	Dynamic Light Scattering
OM	Optical Microscopy
UPV	Ultrasonic Pulse Velocity
CPMG	Carr–Purcell–Meiboom–Gill
CSH	Calcium Silicate Hydrate
PDI	Polydispersity Index
